# Explorative study using ultrasound time-harmonic elastography for stiffness-based quantification of skeletal muscle function

**DOI:** 10.1016/j.zemedi.2024.03.001

**Published:** 2024-03-19

**Authors:** Yang Yang, Mehrgan Shahryari, Tom Meyer, Stephan Rodrigo Marticorena Garcia, Steffen Görner, Mahsa Salimi Majd, Jing Guo, Jürgen Braun, Ingolf Sack, Heiko Tzschätzsch

**Affiliations:** a Department of Radiology, Charité – Universitätsmedizin Berlin, Corporate Member of Freie Universität Berlin and Humboldt-Universität zu Berlin, Charitéplatz 1, 10117 Berlin, Germany; b Institute of Medical Informatics, Charité – Universitätsmedizin Berlin, Corporate Member of Freie Universität Berlin and Humboldt-Universität zu Berlin, Charitéplatz 1, 10117 Berlin, Germany

**Keywords:** Ultrasound Elastography, Musculoskeletal System, Stiffness, Isometric Contraction/Exercise, Time-Harmonic Elastography

## Abstract

Time-harmonic elastography (THE) is an emerging ultrasound imaging technique that allows full-field mapping of the stiffness of deep biological tissues. THE's unique ability to rapidly capture stiffness in multiple tissues has never been applied for imaging skeletal muscle. Therefore, we addressed the lack of data on temporal changes in skeletal muscle stiffness while simultaneously covering stiffness of different muscles.

Acquiring repeated THE scans every five seconds we quantified shear-wave speed (SWS) as a marker of stiffness of the long head (LHB) and short head (SHB) of biceps brachii and of the brachialis muscle (B) in ten healthy volunteers. SWS was continuously acquired during a 3-min isometric preloading phase, a 3-min loading phase with different weights (4, 8, and 12 kg), and a 9-min postloading phase. In addition, we analyzed temporal SWS standard deviation (SD) as a marker of muscle contraction regulation.

Our results (median [min, max]) showed both SWS at preloading (LHB: 1.04 [0.94, 1.12] m/s, SHB: 0.86 [0.78, 0.94] m/s, B: 0.96 [0.87, 1.09] m/s, *p* < 0.001) and the increase in SWS with loading weight to be muscle-specific (LHB: 0.010 [0.002, 0.019] m/s/kg, SHB: 0.022 [0.017, 0.042] m/s/kg, B: 0.039 [0.019, 0.062] m/s/kg, *p* < 0.001). Additionally, SWS during loading increased continuously over time by 0.022 [0.004, 0.051] m/s/min (*p* < 0.01). Using an exponential decay model, we found an average relaxation time of 27 seconds during postloading. Analogously, SWS SD at preloading was also muscle-specific (LHB: 0.018 [0.011, 0.029] m/s, SHB: 0.021 [0.015, 0.027] m/s, B: 0.024 [0.018, 0.037] m/s, *p* < 0.05) and increased by 0.005 [0.003, 0.008] m/s/kg (*p* < 0.01) with loading. SWS SD did not change over loading time and decreased immediately in the postloading phase.

Taken together, THE of skeletal muscle is a promising imaging technique for in vivo quantification of stiffness and stiffness changes in multiple muscle groups within seconds. Both the magnitude of stiffness changes and their temporal variation during isometric exercise may reflect the functional status of skeletal muscle and provide additional information to the morphological measures obtained by conventional imaging modalities.

## 1 Introduction

Elastography is an emerging medical imaging technique that allows in vivo quantification of tissue stiffness for diagnosis and treatment monitoring [1], [2], [3]. Tissue stiffness is particularly relevant in skeletal muscle, where function is directly related to mechanical action, which in turn is associated with marked changes in stiffness. Therefore, ultrasound elastography has been used as a research tool to directly quantify skeletal muscle function under normal and abnormal conditions [4], [5], [6], [7], [8], [9]. However, accurate assessment of skeletal muscle function is still challenging, mainly due to the anisotropy and heterogeneity of muscle anatomy. In addition, muscle function involves different action modes such as isometric and isotonic contraction, where muscle length and force remain constant, respectively. Changes in muscle length can be measured using morphological markers such as muscle thickness, circumference, and manual muscle examination [10], [11], [12]. In contrast, measuring muscle tension requires quantification of force, which in terms of continuum mechanics is expressed as stress (force per unit area) and stiffness (synonymous with stress, assuming unit strain).

Therefore, we here propose time-harmonic elastography (THE) as a novel ultrasound-based elastography technique for measuring muscle function. THE has been developed to generate large-window stiffness maps in deep organs such as the liver [13], [14], [15], [16], [17], [18], [19], [20], [21], [22], [23], spleen [21], kidney [24], [25], pancreas [26], heart [27], [28] and brain [29], [30], [31], [32]. THE could overcome the shortcomings of other elastography techniques such as acoustic radiation force impulse (ARFI)-based methods [33], [34], [35] which are often limited in penetration depth and window size, or magnetic resonance elastography (MRE), which is more time consuming and expensive [36]. THE combines mechanical stimulation by time-harmonic shear waves, as in MRE, with fast ultrasound-based motion encoding, as in ARFI elastography. This allows THE to generate stiffness maps covering the entire field of view of the ultrasound B-mode image with penetration depths up to 13 cm [21]. With its ability to synchronously cover different muscle groups, THE may provide comprehensive muscle assessment in future diagnostic applications. Furthermore, with short repetition times of only a few seconds, THE could be used to monitor temporal stiffness changes over minutes of loading and thus leverage temporal stiffness variation as a functional marker. For example, sustained loading is known to cause superposition of action potentials and recruitment of an additional motor unit. Intrinsic muscular control of action force may result in stiffness changes on different time scales that are potentially detectable when stiffness can be recorded continuously using THE. Therefore, we will use the temporal standard deviation of stiffness measured by THE as a surrogate for the intrinsic and muscle-specific regulation of contraction during sustained exercise.

In this exploratory study, we apply THE to the upper arm muscles to reveal new elastography parameters. We hypothesize that stiffness and standard deviation will be muscle-specific and will increase with load. In addition, we expect stiffness to change in a time-dependent manner during loading. Based on previous work by Kronlage et al., we also expect a time-dependent change in stiffness during postloading [37]. Our goal is to introduce a clinically applicable elastography method that can provide a series of stiffness maps during muscle activity across different muscles. Furthermore, with a view to introducing temporal variation of muscle stiffness as a clinical imaging marker, we specifically analyzed temporal changes in stiffness and stiffness standard deviation in multiple muscles simultaneously. To this end, we studied different flexor muscles of the upper arm during the preload phase (baseline/rest), the loading phase with different weights (contraction) and during the postload phase (recovery) in a group of healthy volunteers.

## 2 Materials and methods

### 2.1 Subjects

The study protocol conformed to the Declaration of Helsinki and was approved by the institutional review board of Charité – Universitätsmedizin Berlin (EA4/040/22). Informed written consent was obtained from all study participants. Inclusion criteria were no history of upper limb trauma within the past 2 years and no muscular or neurologic disease. Given the known sex difference in muscle stiffness, we only included male volunteers in order to investigate a homogeneous study sample [38], [39], [40], [41]. Exclusion criteria were excessive muscle training within the last 24 hours and alcohol intake within the last 24 hours, and eating and drinking within the last 2 hours to avoid possible hydration-induced stiffness changes as recently reported for the liver and brain [21], [29]. All participants were investigated at room temperature.

### 2.2 Time-harmonic elastography

**Figure 1:**
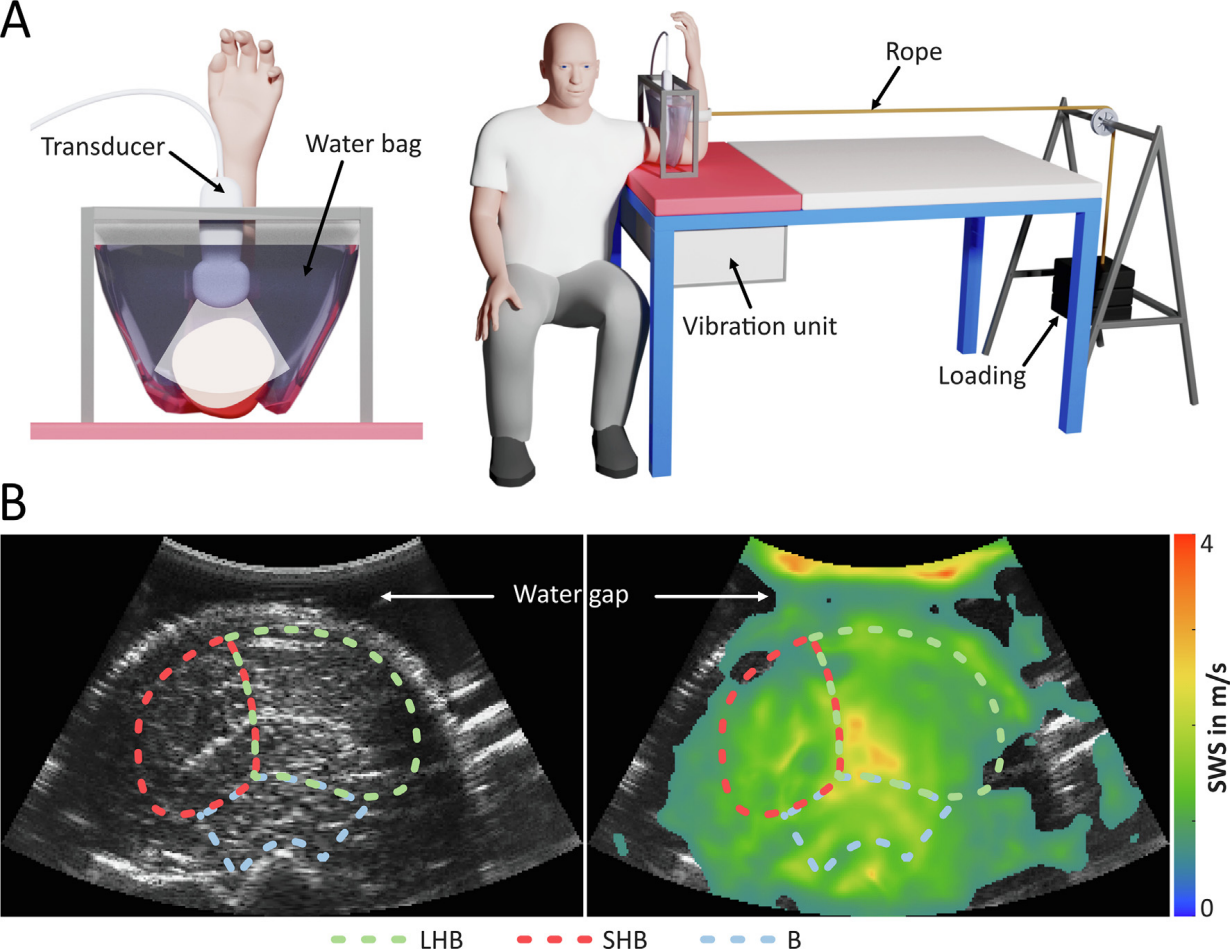
A: Setup of time-harmonic elastography (THE) for the loading phase. Left: The transducer is coupled to the upper arm with a water bag. This avoids external pressure on the muscle and extends the ultrasonic field-of-view. The elbow support for the pre- and postload phases and the transducer stand to avoid unintentional changes of the image section are not shown. Right: Position of the subject on the patient bed with the integrated vibration unit and the cable with the loading weight; B: B-mode image (left) and shear-wave speed (SWS) map (right) of the transverse view of the upper arm with corresponding region of interest (LHB – long head of biceps brachii, SHB – short head of biceps brachii, B – brachialis muscle).

THE uses external harmonic vibration to generate shear waves in the human body, captures the deflection with ultrasound imaging, and finally generates quantitative maps of the underlying tissue stiffness. Harmonic vibration was induced using a patient bed with an integrated loudspeaker (see Fig. 1, GAMPT mbH, Merseburg, Germany). Multifrequency vibration in the frequency range of 27 to 56 Hz was used. Due to the low frequencies, the resulting shear waves penetrated deep into the tissue through the entire muscle group of interest with almost no attenuation. The resulting tissue deflection was captured with a standard ultrasound scanner (Ultrasonix SonixRP, Scottsdale USA) equipped with a convex transducer (C5-2/60, 4 MHz center frequency, 128 elements, 61 mm curvature, 56° field-of-view). Radiofrequency data were acquired over 1 second, with a depth of 8 cm and a frame rate of 80 Hz. During postprocessing, spatially resolved axial tissue displacement between each adjacent frame was calculated from phase differences of radiofrequency data using the Kasai algorithm [42]. Maps of shear-wave speed (SWS) were generated using the wave number-based gradient inversion method k-MDEV [21]. With this method, the wave interference pattern obtained after temporal frequency decomposing via Fourier transformation was spatially bandpass-filtered to suppress the compression wave and noise. Vibration frequencies above the Nyquist frequency were extracted from the aliased frequency bins according to the principle of *controlled aliasing* [21]. Next, the interference pattern was decomposed into 8 plane shear waves using a spatial directional filter. Finally, SWS was calculated using a noise-robust phase gradient method based on the approximate assumption of a linear elastic material, local homogeneity, transverse isotropic tissue, and linear shear-wave frequency dispersion. All post-processing was performed on-site and took less than three seconds. More details can be found in [21].

### 2.3 Setup and study protocol

Muscle stiffness of both arms was measured in 3 phases (3-minute preloading, 3-minute loading, and 9-minute postloading) and repeated 3 times on different days (both arms on the same day with a short break) within two weeks using different loads during the loading phase (4 kg, 8 kg, and 12 kg). The volunteer was sitting on a stool in front of the vibration bed with the elbow placed directly on the vibration unit. During acquisition, the elbow was held at 90° and supported by a stand (three vertical plates to prevent lateral movement and stretching; the stand is not shown in the figure) to maintain a relaxed state (Fig. 1). The wrist was positioned with the palm facing the volunteer and elbow and both shoulders were aligned. Since mechanical muscle properties are known to depend on the angulation of the adjacent joints, particular attention was paid to making sure that volunteers maintained the 90° position of the elbow throughout the investigation. The transducer was mounted on a clamp and fixed in place to avoid inadvertent alteration of the image section by the operator throughout the acquisition period and was placed inside a water bag to avoid pressure on the anterior upper arm (Fig. 1). The imaging slice was adjusted to cover the whole cross-sectional area of three flexor muscles of the upper arm. The ultrasound transducer was placed to capture the largest circumference of the upper arm in the transverse plane. Muscle is approximately a transverse isotropic medium and can be characterized by transverse stiffness and longitudinal stiffness or transverse SWS and longitudinal SWS. In muscle, transverse SWS is lower than longitudinal SWS [6]. In our setup, we used a transverse image section, where SWS is independent of the direction of propagation, and we ensured that we only measured transverse SWS. This setup allowed us to combine the information from all filtered directions, thus enhancing the stability of the final SWS value obtained. This combination is only possible in THE and not in acoustic radiation force impulse (ARFI) imaging-based elastography. We used the measured transverse SWS as a direct surrogate for transverse muscle stiffness. During each phase, 1-second measurements were taken every 5 seconds, corresponding to a total of 36 elastograms and B-mode images each for the preloading and the loading phase (each 3 min) and 108 elastograms and B-mode images for the postloading phase, which was 9 min. For investigation of the muscle during loading, the elbow stand was removed, and a rope was connected to the lower arm at a height of 15 cm from the patient bed to ensure the same angular momentum for all volunteers. The rope was connected to the load using a pulley mounted on a wooden shelf. During the 3-minute loading phase, the volunteer was asked to actively maintain a 90° elbow angle. For measurement of postloading muscle stiffness, the load was removed while the 90° elbow angle was maintained by the stand.

### 2.4 Data processing

**Figure 2:**
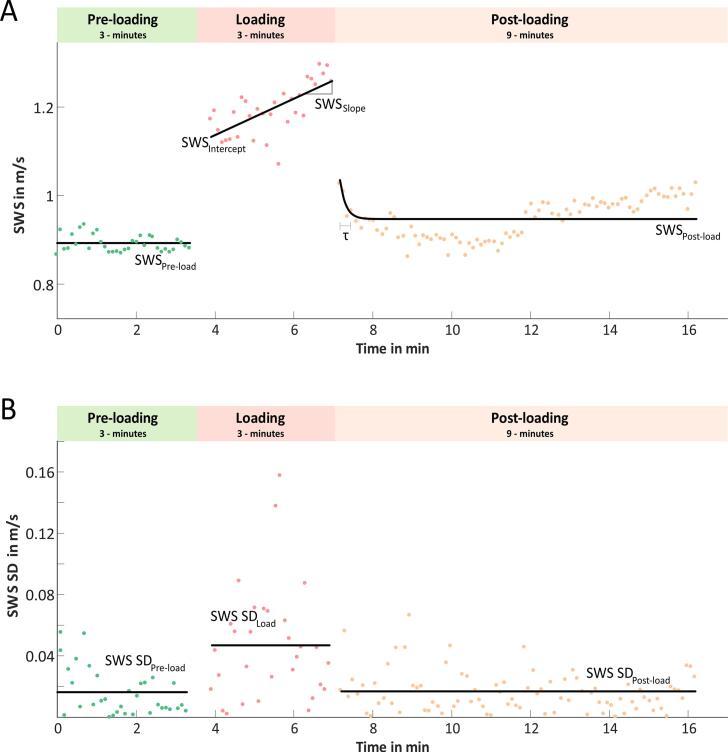
Shear-wave speed (SWS) data and temporal standard deviation (SD) of SWS over the three phases (3-min preload, 3-min load, and 9-min postload) with corresponding fits (black lines). Shown are data for the long head of the biceps brachii of the left arm of one subject at 12 kg load. (Relaxation time τ).

For both arms, regions of interest (ROIs) were manually drawn in three anatomical regions (long head of biceps brachii, LHB; short head of biceps brachii, SHB; brachialis muscle, B, see Fig. 1 B) for each of the three loading states and experimental phases based on the B-mode image, resulting in a total of 3,240 ROIs for each volunteer (2 × 3 × 3 × (36 + 36 + 108)). The ROI covered the entire anatomical cross-section of the muscle visible in the B-mode. The stability of the measurements was further increased by spatial averaging, i.e. taking into account the average values over the muscle ROI. Further processing was done separately for each volunteer, loading phase, and muscle (example in Fig. 2).

During preloading, SWS was averaged over the entire 3-minute phase to obtain the ${\mathrm{SWS}}_{preload}$ value. For the loading phase, we modelled the SWS change over time *t* with a linear regression model (equation (1), yielding ${\mathrm{SWS}}_{intercept}$ and temporal ${\mathrm{SWS}}_{slope}$. The robust linear model approach (1^st^ order regression) was chosen based on the results of a preliminary analysis of the behavior of our data over time.

(1) $$SWS\left(t\right)={\mathrm{SWS}}_{intercept}+{\mathrm{SWS}}_{slope}\cdot t$$

The temporal course of SWS in the postloading phase was modeled as an exponential decrease (equation (2). This yielded the values of relaxation time τ and final SWS saturation ${\mathrm{SWS}}_{postload}$. The exponential decay model was chosen because it is the most robust and fundamental relaxation function, which is also supported by the results of Kronlage et al. [37].

(2) $$SWS\left(t\right)={\mathrm{SWS}}_{relaxation}\cdot {\text{e}}^{-\frac{t}{\tau }}+{\mathrm{SWS}}_{postload}$$

Data processing for SWS is illustrated for one volunteer in Fig. 2 A. To analyze the temporal variation of SWS, the time-resolved standard deviation (SD) was derived from the acquired time-series of SWS. Therefore, we calculated SD(t) as the difference between two adjacent time points according to Popovic and Thomas [43]. With this equation, the standard deviation could be calculated time-resolved instead of determining an average value over the entire measurement time. Therefore, the underlying temporal modulation of the SWS could be separated.

(3) $$SWSSD\left(t_{i}\right)=\frac{1}{\sqrt{2}}\;\left|\frac{SWS\left(t_{i+1}\right)\;-\;SWS\left(t_{i}\right)}{t_{i+1}\;-\;t_{i}}\right|\;.$$

In preliminary test experiments, we did not visually observe a temporal change in SWS SD within each of the three phases. Therefore, we calculated only the corresponding phase-averaged value by temporal averaging, which yielded ${\mathrm{SWSSD}}_{preload}$, and ${\mathrm{SWSSD}}_{postload}$. Data processing for SWS SD in one volunteer is illustrated in Fig. 2 B.

### 2.5 Maximum voluntary contraction

Maximum voluntary contraction (MVC) was used to standardize the applied load relative to the maximum individual contraction force each subject could achieve. MVC was measured using the same setup as for THE except that the load was replaced by a dynamometer (electronic hand dynamometer, model EH101). Each volunteer performed maximum contraction for 5 seconds with the elbow at 90° flexion. This measurement was repeated three times and the highest value was used. MVC values of both arms were averaged, and relative values were calculated for each of the three different loads. Since we used the same load for all subjects, we present relative MVC to demonstrate the comparability of our THE results.

### 2.6 Statistics

As no laterality in is known from published data and the Wilcoxon signed-rank test showed no significant differences between the right and left arm for our SWS and SD data, SWS values of both arms were averaged [44], [45], [46], [47]. Because of the small number of volunteers investigated in our experiment, the nonparametric Friedman’s test was used to test for differences between muscles for all SWS parameters (${\mathrm{SWS}}_{preload}$, ${\mathrm{SWS}}_{intercept}$ over loading, ${\mathrm{SWS}}_{slope}$, *τ,* ${\mathrm{SWS}}_{postload}$). If a significant difference between muscles was found, a Conover post-hoc test was conducted for the different muscles. In addition, we also used the Friedman’s test to test differences in ${\mathrm{SWS}}_{intercept}$ between different loadings. A Conover post-hoc test was conducted for the different loadings, and linear regression of ${\mathrm{SWS}}_{intercept}$ over load was fitted. The statistical tests were performed analogously for SWS SD: a Friedman’s test was used to test parameters (${\mathrm{SWSSD}}_{preload}$, ${\mathrm{SWSSD}}_{load}$ over loading, ${\mathrm{SWSSD}}_{postload}$) for differences between muscles. If a significant difference between muscles was found, a Conover post-hoc test was conducted. We also used the Friedman’s test to test differences in ${\mathrm{SWSSD}}_{load}$ between different loadings and a Conover post-hoc test was conducted for the different loadings and a linear regression of ${\mathrm{SWSSD}}_{load}$ over load was fitted.

A Wilcoxon signed-rank test was used to test for i) SWS changes over loading time (${\mathrm{SWS}}_{slope}$), ii) relaxation time *τ,* iii) SWS differences between pre- and postloading (${\mathrm{SWS}}_{preload}-{\mathrm{SWS}}_{postload}$), iv) SWS SD changes across loading weights, and v) SWS SD differences between pre- and postloading (${\mathrm{SWSSD}}_{preload}-{\mathrm{SWSSD}}_{postload}$). Repeatability coefficient was calculated from repeated SWS and SWS SD preload measurements for both sides and all loads. Statistical analysis was performed in R (version 4.1.1; R-Foundation, Vienna, Austria) using R-Studio (version 2021.09.0; RStudio, PBC, Boston, MA). Values are reported as median and range unless stated otherwise.

## 3 Results

THE was successful in all participants, as indicated by mean shear wave amplitudes >2 µm within the ROI, which has been considered sufficient in the literature [29]. Median [min, max] ROI areas for the preloading phase were 5.5 [2.8, 9.4] cm^2^ for LHB, 2.9 [1.7, 4.8] cm^2^ for SHB and 1.8 [1.3, 3.2] cm^2^ for B. All volunteers were right-handed and no volunteer was excluded. The volunteers were 30 [27], [51] years old and had a body mass index (BMI) of 22 [19], [28] kg/m^2^. MVC was 43 [30], [49] kg for both arms with higher values in the right (45 [33], [49] kg) than in the left arm (41 [27], [49] kg, *p* = 0.0039). Relative MVC values at different loads were 9 [8 13] % for 4 kg, 19 [16 27] % for 8 kg, and 28 [25,40] % for 12 kg. All results are compiled in Table 1.

**Table 1:** Age, body mass index (BMI), and maximum voluntary contraction (MVC) with percentage MVC at the three different loads investigated for each volunteer.

Volunteer	Age	BMI	MVC			MVC corresponding		
			right	left	mean	4 kg	8 kg	12 kg
#	years	kg/m^2^	kg	kg	kg	%	%	%
1	30	28	40	39	39	10	20	30
2	36	20	44	37	40	10	20	30
3	29	23	38	37	38	11	21	32
4	51	22	48	43	45	9	18	27
5	30	21	48	42	45	9	18	27
6	30	22	33	27	30	13	27	40
7	29	24	41	39	40	10	20	30
8	30	19	47	47	47	9	17	26
9	30	25	48	45	46	9	17	26
10	27	23	49	49	49	8	16	25

Min	27	19	33	27	30	8	16	25
Median	30	22	45	41	43	9	19	28
Max	51	28	49	49	49	13	27	40

### 3.1 SWS

**Figure 3:**
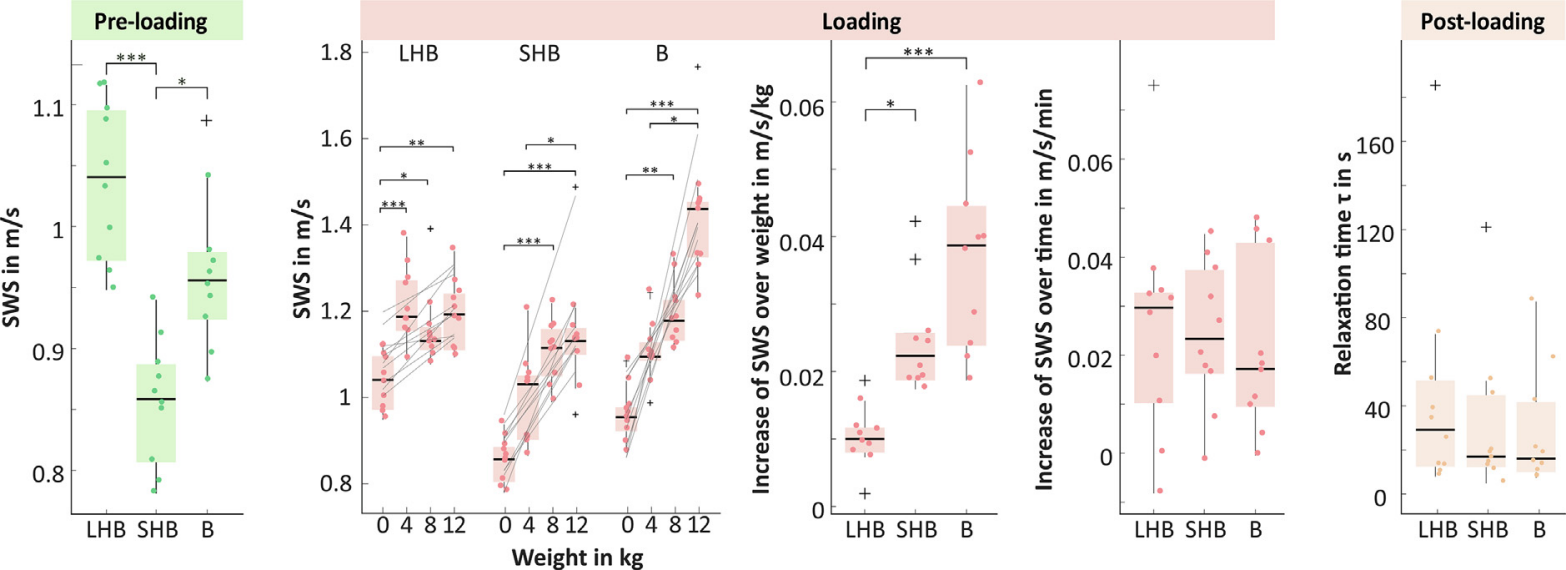
Shear-wave speed (SWS) parameters for the three phases investigated: preloading, loading, and postloading. Results are shown separately for the long head of biceps brachii (LHB), short head of biceps brachii (SHB), and brachialis muscle (B).

During the preloading phase, ${\mathrm{SWS}}_{preload}$ of all volunteers were 1.04 [0.94, 1.12] m/s for LHB, 0.86 [0.78, 0.94] m/s for SHB, and 0.96 [0.87, 1.09] m/s for B. A difference between individual muscles was observed between LHB and SHB (*p* < 0.001) and between SHB and B (*p* < 0.05). In the loading phase, we observed a significant difference in ${\mathrm{SWS}}_{intercept}$ between the loadings. Therefore, linear regression of SWS over loading weight yielded an SWS increase of 0.010 [0.002, 0.019] m/s/kg for LHB (1.0  % per kg), 0.022 [0.017, 0.042] m/s/kg for SHB (2.6% per kg), and 0.039 [0.019, 0.062] m/s/kg for B (4.1% per kg). Here, significant differences were observed between LHB and SHB (*p* < 0.05) and between LHB and B (*p* < 0.001). During the loading phase, the ${\mathrm{SWS}}_{slope}$ over time was not different between the different loading weights investigated. The ${\mathrm{SWS}}_{slope}$ were 0.030 [−0.008, 0.075] m/s/min for LHB (2.6–2.8% per min), 0.023 [−0.002, 0.045] m/s/min for SHB (2.0–2.4% per min), and 0.017 [0.000, 0.048] m/s/min for B (1.2–1.5% per min). The root mean square error for the loading (equation (1) were 0.04 [0.02, 0.13] m/s for LHB, 0.04 [0.02, 0.13] m/s for SHB and 0.05 [0.02, 0.13] m/s for B. There was no difference between the muscles, and the averaged value was 0.022 [0.004, 0.051] m/s/min and was different from zero (*p* < 0.01). In the postloading phase, relaxation time *τ* was not different between the loading weights. Relaxation time *τ* were 17 [5, 121] s for LHB, 16 [7, 87] s for SHB, and 29 [8, 185] s for B. Relaxation time was also not different between the muscles, and the averaged value was 27 [9], [73] seconds. The root mean square error for the postloading fit (equation (2) were 0.02 [0.01, 0.03] m/s for LHB, 0.02 [0.01, 0.04] m/s for SHB, and 0.02 [0.01, 0.03] m/s for B. ${\mathrm{SWS}}_{postload}$ was not different between loading weights or between muscles. The average across muscles and loads was higher (difference = 0.036 m/s) than ${\mathrm{SWS}}_{preload}$ (*p <* 0.01). SWS results are summarized in Table 2 and shown in Fig. 3. The repeatability coefficient of SWS was 0.16 m/s for LHB, 0.18 m/s for SHB and 0.24 m/s for B.

**Table 2:** Shear**-**wave speed (SWS) parameters for the three phases (preloading, loading, and postloading) measured by THE. Mean values (confidence Interval) are presented separately for long head of biceps brachii (LHB), short head of biceps brachii (SHB), and brachialis muscle (B). Please note that the increase over loading weight is derived from a single linear regression over all loadings and therefore does not have a confidence interval.

	**SWS**											
	**Pre-loading**			**Increase over loading weight**			**Increase over loading time**			**Relaxation time** τ		
	**LHB**	**SHB**	**B**	**LHB**	**SHB**	**B**	**LHB**	**SHB**	**B**	**LHB**	**SHB**	**B**
**#**	**m/s**	**m/s**	**m/s**	**m/s/kg**	**m/s/kg**	**m/s/kg**	**m/s/min**	**m/s/min**	**m/s/min**	**s**	**s**	**s**
1	1.12 (1.07 1.16)	0.91 (0.87 0.96)	0.98 (0.94 1.01)	0.007	0.019	0.024	0.019 (−0.006 0.044)	−0.002 (−0.037 0.034)	0.020 (−0.018 0.058)	121 (−2 49)	42 (−5 17)	73 (12 133)
2	1.05 (1.01 1.09)	0.81 (0.78 0.84)	1.04 (0.97 1.11)	0.011	0.037	0.022	0.037 (−0.007 0.081)	0.045 (0.007 0.083)	0.011 (−0.011 0.033)	16 (7 25)	14 (11 17)	33 (19 48)
3	0.96 (0.92 1.00)	0.86 (0.82 0.91)	0.89 (0.85 0.94)	0.011	0.021	0.045	0.031 (0.008 0.054)	0.020 (−0.025 0.065)	0.048 (0.023 0.072)	12 (7 17)	13 (7 18)	185 (−3 31)
4	1.10 (1.02 1.17)	0.85 (0.77 0.93)	0.92 (0.84 1.01)	0.012	0.026	0.028	0.000 (−0.014 0.014)	0.007 (−0.022 0.036)	0.004 (−0.052 0.060)	5 (0 10)	61 (−32 154)	13 (6 20)
5	0.97 (0.91 1.04)	0.85 (0.81 0.90)	0.96 (0.88 1.04)	0.009	0.024	0.040	0.033 (0.023 0.043)	0.027 (0.010 0.044)	0.043 (0.014 0.072)	11 (6 15)	7 (3 12)	10 (5 14)
6	1.12 (1.07 1.16)	0.87 (0.82 0.93)	0.94 (0.88 1.00)	0.009	0.019	0.040	0.028 (−0.015 0.071)	0.017 (−0.011 0.046)	0.018 (−0.027 0.062)	14 (8 20)	87 (−61 236)	25 (9 41)
7	1.03 (1.00 1.06)	0.89 (0.78 1.00)	1.09 (1.00 1.17)	0.002	0.017	0.019	0.010 (−0.022 0.042)	0.040 (0.026 0.054)	0.009 (−0.021 0.040)	19 (7 32)	10 (4 16)	51 (−18 121)
8	0.95 (0.90 0.99)	0.78 (0.75 0.81)	0.87 (0.79 0.95)	0.016	0.025	0.052	−0.008 (−0.035 0.019)	0.016 (−0.009 0.041)	0.017 (−0.003 0.036)	18 (10 26)	20 (0 41)	12 (9 16)
9	1.00 (0.96 1.03)	0.79 (0.73 0.85)	0.97 (0.89 1.05)	0.008	0.019	0.038	0.032 (−0.003 0.067)	0.037 (0.003 0.072)	0.000 (−0.038 0.037)	51 (−17 120)	7 (4 11)	8 (4 12)
10	1.09 (1.04 1.13)	0.94 (0.92 0.96)	0.95 (0.89 1.01)	0.019	0.042	0.062	0.075 (0.050 0.100)	0.031 (−0.023 0.086)	0.045 (0.009 0.082)	45 (−4 94)	18 (10 26)	38 (3 73)

Min	0.95	0.78	0.87	0.002	0.017	0.019	−0.008	−0.002	0.000	5	7	8
Median	1.04	0.86	0.96	0.010	0.022	0.039	0.030	0.023	0.017	17	16	29
Max	1.12	0.94	1.09	0.019	0.042	0.062	0.075	0.045	0.048	121	87	185

### 3.2 SWS SD

**Figure 4:**
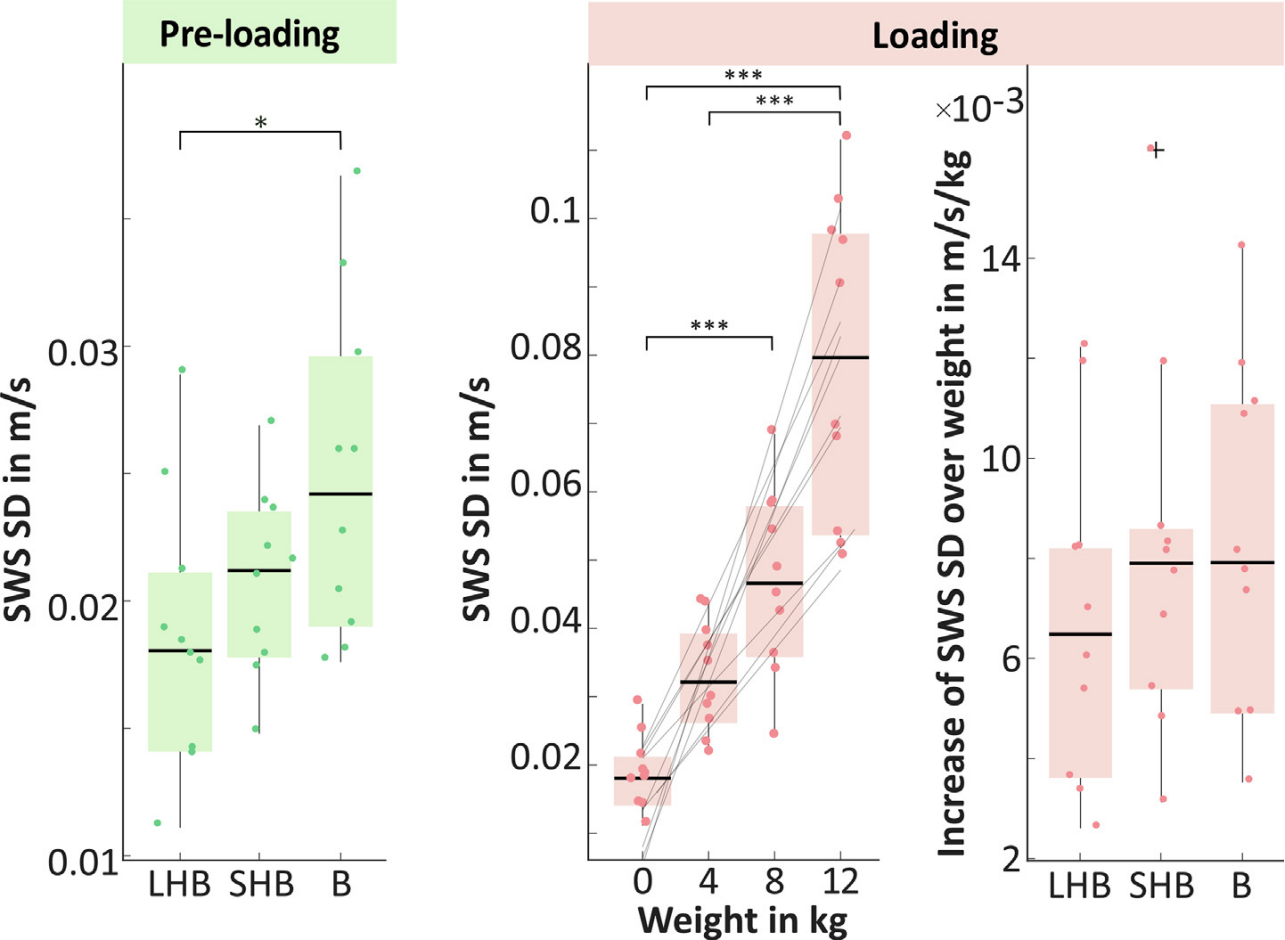
SWS standard deviation (SD) parameters for two phases: preloading and loading. Results are shown separately for long head of biceps brachii (LHB), short head of biceps brachii (SHB), and brachialis (B).

For the preloading phase, ${\mathrm{SWSSD}}_{preload}$ were 0.018 [0.011, 0.029] m/s for LHB, 0.021 [0.015, 0.027] m/s for SHB, and 0.024 [0.018, 0.037] m/s for B. A difference between the individual muscles was observed between LHB and B (*p* < 0.05). In the loading phase, we found a significant difference between the loadings for ${\mathrm{SWSSD}}_{load}$. Therefore, linear regression of SWS SD over loading weight yielded an SWS SD increase of 0.004 [0.001, 0.008] m/s/kg for LHB (22% per kg), 0.005 [0.002, 0.010] m/s/kg for SHB (21% per kg), and 0.005 [0.002, 0.009] m/s/kg for B (24% per kg). There was no difference between the muscles, and the averaged value was 0.005 [0.003, 0.008] m/s/kg and was different from zero (*p <* 0.01). In contrast to SWS for SWS SD, we observed an immediate decrease from loading to postloading. In the postloading phase, $\mathrm{SWS}{\mathrm{SD}}_{postload}$ was not different between loading weights or between muscles. The average across muscles and loads was not different from ${\mathrm{SWSSD}}_{preload}$ (*p* = 0.77). SWS SD results are summarized in Table 3 and shown in Fig. 4. The repeatability coefficient of SWS SD was 0.16 m/s for LHB, 0.16 m/s for SHB and 0.21 m/s for B.

**Table 3:** Temporal SWS standard deviation (SD) parameters for the three phases (preloading, loading, and postloading) measured by THE. Mean values (confidence Interval) are presented separately for long head of biceps brachii (LHB), short head of biceps brachii (SHB), and brachialis muscle (B). Please note that the increase over loading weight is derived from a single linear regression over all loadings and therefore does not have a confidence interval.

	**SWS SD**					
	**Pre-loading**			**Increase over loading weight**		
	**LHB**	**SHB**	**B**	**LHB**	**SHB**	**B**
**#**	**m/s**	**m/s**	**m/s**	**m/s**	**m/s**	**m/s**
1	0.018 (0.014 0.021)	0.019 (0.012 0.025)	0.020 (0.014 0.027)	0.002	0.003	0.003
2	0.018 (0.015 0.020)	0.015 (0.013 0.017)	0.033 (0.026 0.040)	0.002	0.005	0.002
3	0.014 (0.011 0.017)	0.018 (0.014 0.022)	0.018 (0.014 0.022)	0.005	0.010	0.009
4	0.018 (0.011 0.026)	0.017 (0.015 0.019)	0.018 (0.015 0.021)	0.008	0.005	0.007
5	0.014 (0.010 0.018)	0.021 (0.016 0.026)	0.023 (0.017 0.028)	0.001	0.002	0.004
6	0.021 (0.016 0.027)	0.027 (0.022 0.032)	0.026 (0.018 0.034)	0.004	0.003	0.005
7	0.019 (0.012 0.026)	0.024 (0.017 0.030)	0.026 (0.018 0.034)	0.003	0.005	0.003
8	0.011 (0.009 0.013)	0.022 (0.013 0.030)	0.019 (0.012 0.026)	0.005	0.007	0.007
9	0.025 (0.017 0.032)	0.022 (0.019 0.025)	0.037 (0.028 0.045)	0.007	0.004	0.005
10	0.029 (0.026 0.032)	0.024 (0.019 0.029)	0.030 (0.026 0.033)	0.004	0.005	0.007

Min	0.011	0.015	0.018	0.001	0.002	0.002
Median	0.018	0.021	0.024	0.004	0.005	0.005
Max	0.029	0.027	0.037	0.008	0.010	0.009

## 4 Discussion

To the best of our knowledge, this is the first THE study in skeletal muscle to provide stiffness maps across the entire upper arm muscle anatomy within seconds. Using this promising technique, we showed that stiffness (SWS) i) varied across upper arm flexors in preload, ii) increased with loading weight and duration, and iii) recovered with a prolonged decay in the postload phase (τ). In addition, we found that temporal changes in stiffness (SWS SD) iv) varied across muscles during the preload phase and v) increased with loading weight.

Several ultrasound elastography studies have shown that muscle stiffness increases with loading [48], [49], [50], [51], depends on the angulation of adjacent joints [6], [52], [53], [54], [55], [56], and, due to anisotropy, is higher when measured parallel to muscle fibers [6], [57]. Furthermore, it has been reported that muscle stiffness exhibits a relaxation behavior of different time scales after loading that is affected by muscular disorders [37], [58].

### 4.1 SWS

The high penetration depth of THE of 8 cm allowed us to simultaneously measure the stiffness of three muscles in the upper arm. The differences in baseline stiffness across muscles might be related to muscle geometry and prestretch resulting from the skeletal lever system, which exerts inertial forces that depend on individual anatomy [59], [60]. This interpretation is supported by the findings of Gennisson et al*.* showing that elbow flexion affects muscle stiffness in longitudinal views as well [6]. The literature on the transverse shear modulus of the human upper arm muscle is very sparse, which limits the comparability of our data with those from ground-truth methods. Nevertheless, our SWS values in the preloading phase are comparable to stiffness values reported for the biceps brachii (1.27 ± 0.06 m/s at 50 Hz [6]; 2.22 ± 0.18 m/s at 75–126 Hz [61]) and for different leg muscles (0.98–1.13 m/s at 40 Hz in the tibialis anterior, tibialis posterior, peroneus, extensor digitorum longus, soleus, and gastrocnemius [62]; 4.83–7.68 m/s in the rectus femoris, vastus lateralis, and medial and lateral gastrocnemius [63]; 1.12–1.15 m/s at 50 Hz in the tibialis anterior, soleus, and medial gastrocnemius [64]; 0.81 m/s at 60 Hz in the tibialis anterior, soleus, and medial gastrocnemius [65]; 0.95–1.12 m/s at 30–60 Hz in the tibialis anterior, soleus, and gastrocnemius [66]).

During the loading phase, we found an SWS increase of 1–4% per kilogram, which is consistent with findings reported by other investigators [6], [62]. However, Gennisson et al. used slightly higher vibration frequencies while Schrank et al. did not control the muscle force. The load-induced increase in muscle stiffness is associated with the formation of myosin-actin bridges during muscle contraction [59]. In addition, there is an increase in perfusion with increasing load [67], which might have also contributed to higher muscle stiffness during loading. We observed a notable muscle-specific increase in stiffness during loading, with LHB being stiffer than SHB in the preload phase, but softer than SHB at 12 kg loading. This observation might be explained by unequal load sharing between the muscles, in which functional units distribute the load differently among the acting muscles [68].

We also found a temporal increase in SWS of 1–3% per minute. This result could be attributable to the fact that venous walls are gradually compressed during contraction, causing a continuous accumulation of metabolites, which are known to influence muscle stiffness. [69], [70].

In the postloading phase, we observed a prolonged decay of stiffness similar to [37]. However, we found a significantly longer relaxation time than Kronlage et al. (27 seconds versus 1 second) where the studied muscles were activated for only 5 seconds while in our study muscle contraction was induced for 3 min. Therefore, the difference in relaxation times between the two studies might be attributable to differences in the fiber recovery time, metabolic activity [59], [71] or pre-stretched connective tissue which exhibits pronounced hysteresis [72]. Furthermore, the offset of 3–4% elevated stiffness 9 min after loading in our study might be explained by a delayed reduction in muscle perfusion, which is known to decrease relatively slowly within seconds after loading [67]. Other studies measuring longitudinal stiffness in the upper arm observed an elevated stiffness of 9–35% [73] and 22% [74] 15 min after exercise and up to 24% 1 h after exercise [75].

### 4.2 SWS SD

We interpret the observed temporal variation of SWS during muscle contraction, quantified by SWS SD, as an intrinsic muscle activity function that maintains constant mechanical forces under sustained load. It is known that isometric muscle contraction requires active neuronal control to ensure a constant muscle force [59]. Therefore, additional motor units are recruited as muscle load increases [67], [68], potentially leading to increased stiffness variability. This may explain our observation of a 21–24% increase in SWS SD per kilogram of load. The complex counterbalancing mechanism of muscle motor units has been reported to cause spatial and temporal force variations in the frequency range of 10–40 Hz [76]. Such force variations could lead to stiffness changes detectable by THE, despite the temporal averaging over one second performed by THE. At a THE frame rate of 80 Hz, stiffness changes detected within a temporal averaging of 1 second are smaller by a factor of $1/\sqrt{80}$ as indicated by the standard error of the mean. Consistent with our findings of increased SWS SD with exercise, electromyography (EMG) studies have found an increase in measured electrical amplitude with load [77], [78]. Our preliminary notion of SWS SD as a potential stiffness-specific surrogate for EMG activity is supported by studies in relaxed muscles that showed significant EMG variation between muscles [79], similar to what we found based on SWS SD measured by THE.

### 4.3 Limitations

The main limitations of our study are the small number of volunteers included and the use of only three different loading weights. Since we investigated only male volunteers, results may be different in women, as suggested by Eby et al. [38]. Possible sources of error that may have affected our results include the training status of the volunteers, transducer position or operator dependency. While we were able to control transducer position with the rack shown in Fig. 1, we did not analyze operator dependencies and individual training conditions in this study. Prior to diagnostic applications, THE in muscle requires standardized reference values that take into account age groups, sex and training conditions, as well as possible operator-induced variability. In addition, the clinical relevance of the new elastography parameter should be investigated in further studies. A technical limitation is that we focused on transverse stiffness to achieve greater measurement stability by combining all shear-wave propagation directions. This would not have been possible in a view parallel to the muscle fibers, where shear waves propagate with different speeds corresponding to longitudinal and transverse wave speeds [61]. Other studies have shown that the load-induced stiffness increase is more pronounced in longitudinal orientation [6].

### 4.4 Conclusion

This is the first ultrasound-based THE study investigating full-field elastography of human skeletal muscle. Using a 5-second repetition time, we continuously quantified the progression of stiffness variation (indicated by SWS) and contraction regulation (indicated by SWS SD) simultaneously for different muscles of the upper arm over several minutes at baseline as well as during isometric contraction and subsequent relaxation. Our results demonstrate a number of fundamental physiological properties of skeletal muscle function, including variation in SWS and SWS SD across muscles, continuous increase in SWS during sustained contraction, and increase in SWS SD with load. These parameters have potential value for diagnostic applications of muscle elastography and comprehensive characterization of muscle function based on in vivo tissue stiffness.

## Declaration of competing interest

The authors declare that they have no known competing financial interests or personal relationships that could have appeared to influence the work reported in this paper.
